# Supplementary material for: Association between depressive symptoms and cognitive–behavioural therapy receipt within a psychosis sample: a cross-sectional study
Source: BMJ Open. 2022 May 10;12(5):e051873. doi: 10.1136/bmjopen-2021-051873 (PMC9092128; doi:10.1136/bmjopen-2021-051873)
Supplement: Supplementary data [file bmjopen-2021-051873supp001.pdf]

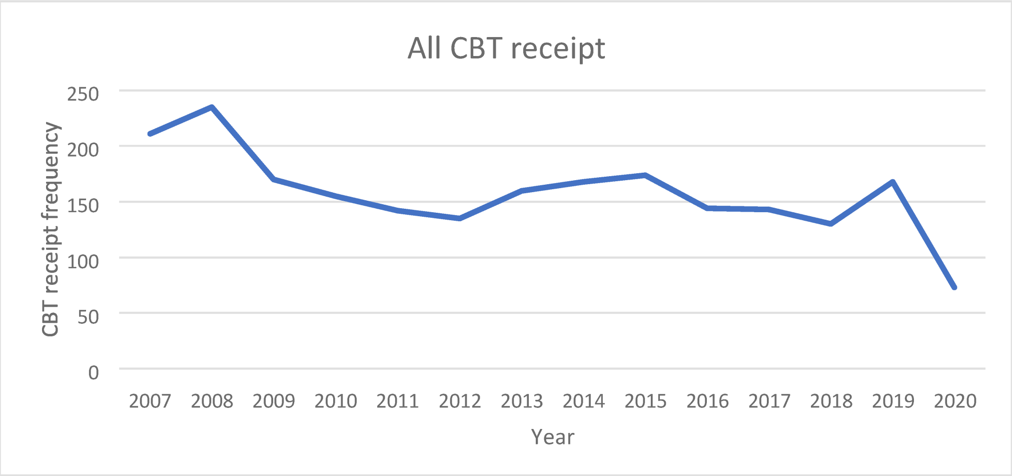

Supplementary figure

Graph demonstrating the frequency of general CBT receipt (CBT receipt prior to diagnosis and recorded CBTp receipt post diagnosis) per year of extraction period.
